# AEPF: Attention-Enabled Point Fusion for 3D Object Detection

**DOI:** 10.3390/s24175841

**Published:** 2024-09-09

**Authors:** Sachin Sharma, Richard T. Meyer, Zachary D. Asher

**Affiliations:** Department of Mechanical and Aerospace Engineering, Western Michigan University, 1903 West Michigan Ave, Kalamazoo, MI 49008, USA; sachin.sharma@wmich.edu (S.S.); zach.asher@wmich.edu (Z.D.A.)

**Keywords:** 3D object detection, sensor fusion, autonomous vehicles, LiDAR, camera

## Abstract

Current state-of-the-art (SOTA) LiDAR-only detectors perform well for 3D object detection tasks, but point cloud data are typically sparse and lacks semantic information. Detailed semantic information obtained from camera images can be added with existing LiDAR-based detectors to create a robust 3D detection pipeline. With two different data types, a major challenge in developing multi-modal sensor fusion networks is to achieve effective data fusion while managing computational resources. With separate 2D and 3D feature extraction backbones, feature fusion can become more challenging as these modes generate different gradients, leading to gradient conflicts and suboptimal convergence during network optimization. To this end, we propose a 3D object detection method, Attention-Enabled Point Fusion (AEPF). AEPF uses images and voxelized point cloud data as inputs and estimates the 3D bounding boxes of object locations as outputs. An attention mechanism is introduced to an existing feature fusion strategy to improve 3D detection accuracy and two variants are proposed. These two variants, AEPF-Small and AEPF-Large, address different needs. AEPF-Small, with a lightweight attention module and fewer parameters, offers fast inference. AEPF-Large, with a more complex attention module and increased parameters, provides higher accuracy than baseline models. Experimental results on the KITTI validation set show that AEPF-Small maintains SOTA 3D detection accuracy while inferencing at higher speeds. AEPF-Large achieves mean average precision scores of 91.13, 79.06, and 76.15 for the car class’s easy, medium, and hard targets, respectively, in the KITTI validation set. Results from ablation experiments are also presented to support the choice of model architecture.

## 1. Introduction

Three-dimensional object detection remains one of the most critical tasks within the perception subsystem for various applications such as autonomous driving, robotics, drone navigation, and augmented reality [1]. The goal of 3D object detection is to predict the location and classes of the objects in the scene and localize them with respect to some known reference. Safety-critical robotic systems require highly accurate information about an object’s depth, position, and volume in a scene for accurate perception. Advancements in computer vision technology have resulted in highly effective 2D object detectors that deliver excellent results using only image data [2,3], but since camera data are 2D by nature and stereo cameras have limited depth detection range, these detectors are unable to provide accurate depth and spatial positioning of objects. Therefore, data from sensors like LiDAR and RADAR is often fused with camera images to provide the high accuracy needed for 3D object detection.

Cameras provide images as an array of pixels where each pixel has three color channels. Although cameras offer rich semantic information about a scene, they inherently lack the ability to directly capture its 3D structural data, and depth information estimated from images typically contains significant errors [1]. Existing literature on camera-based 3D object detectors [4,5,6] demonstrates lower performance primarily due to imprecise depth estimation. On the other hand, LiDARs can provide accurate depth and geometric information via point clouds but are usually sparse due to factors such as small object sizes, long distances between objects, or occlusion situations. Despite achieving competitive performance on 3D detection benchmarks, LiDAR-based 3D object detectors [7,8,9,10,11] struggle under such inclement conditions because of insufficient context to distinguish sparse distant regions.

Several multi-modal sensor fusion methods [12,13,14,15,16,17,18] have been proposed in the literature to improve 3D object detection by utilizing geometric and semantic information from images and point clouds. Three distinct groups of deep-learning-based multi-modal sensor fusion exist: early (data-level), middle (feature-level), and late fusion (decision-level). In the early fusion methods [17,19], raw sensory inputs are fused to help the network learn a joint representation. For example, the authors in [17] performed xdata-level fusion by complementing additional semantic information to the LiDAR-only detection pipeline. At the decision-making stage, late-fusion-based methods [14] process sensory information from different sensor modalities separately and fuse the output at the decision level. With middle-fusion-based methods [12,13,16,18,20], individual features are extracted from multi-modal inputs, and then an intermediate stage is used to learn joint representations. Although feature level fusion methods [12,16,21,22] have shown remarkable success in 3D object detection benchmarks [23,24], the extensive research focus is still on determining at what stage do the features need to be fused. Methods like [12,13,16] combine semantic and geometric features towards the end of both modalities. Coarse-grained features from individual modalities are fused to regress 3D bounding boxes. Extracting coarse-grained features from both modalities requires a higher training and inference cost. These networks also fail to learn shared features between modalities early. Feature-fusion methods such as ref. [18,25] combine features at an earlier stage. The authors in [18] conducted voxel-level fusion by projecting non-empty voxels to the image, allowing them to extract image features for every voxel.

**Figure 1:**
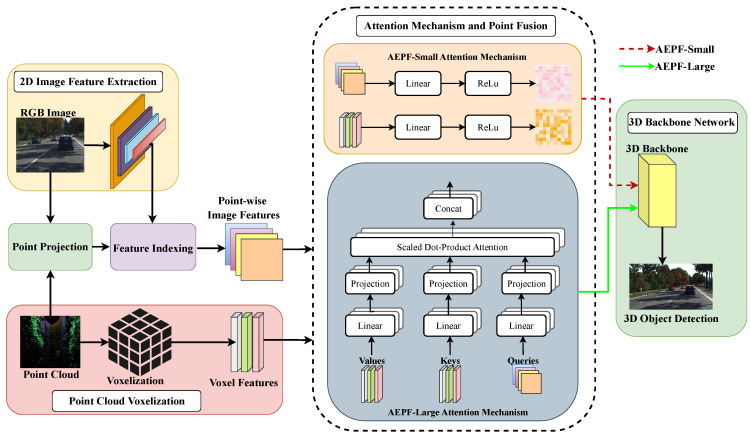
Architecture for AEPF: Attention-Enabled Point Fusion for 3D object detection. Blocks illustrate processes from Section 3.1, Section 3.2, Section 3.3 and Section 3.4. Attention mechanisms for AEPF-Small and AEPF-Large are also shown.

Feature fusion at early stages between different modalities has the most significant opportunity for cross-modal interaction. Given a camera image and a corresponding LiDAR point cloud of a scene, can an early feature-fusion-based method be created that uses the most prominent features from individual modalities and outputs 3D bounding boxes with improved accuracy? The overall hypothesis is that image and point cloud features can be fused while selecting the most prominent features from individual modalities pre-fusion with attention mechanisms to enhance 3D detection results. To this end, a novel multi-modal and multi-class 3D object detector named Attention-Enabled Point Fusion (AEPF) for 3D object detection is proposed as shown in Figure 1, which takes in images and point cloud data as inputs and outputs 3D bounding boxes after passing through the attention-enabled sensor fusion layers. Two AEPF model variants are proposed: AEPF-Small (AEPF-S) and AEPF-Large (AEPF-L). AEPF-S employs attention mechanisms for both image and point cloud features before fusion, while AEPF-L utilizes multi-head self-attention and uses image features to highlight important point cloud features before fusion. The contributions of this work are as follows:

1. A novel feature fusion methodology—AEPF is proposed for 3D object detection. The proposed feature fusion methodology utilizes an attention mechanism to highlight important features within individual sensor modalities.
2. Two object detection variants based on AEPF architecture are presented and validated. AEPF-S maintains the accuracy of state-of-the-art (SOTA) algorithms while inferencing at higher speeds. AEPF-L obtains competitive results in the overall 3D mean average precision (mAP) category on the KITTI validation set and is intended for scenarios prioritizing higher accuracy with sufficient computational resources available.
3. The proposed 3D object detection method is validated after extensive experiments in the KITTI dataset [23]. The effectiveness of key network design components is verified by performing ablation studies.

An overview of this paper is as follows. Section 2 presents a literature review on current SOTA 3D object detection methods across different sensor modalities, the architecture for the proposed 3D object detection method is described in Section 3, experimental results on KITTI data and ablation studies are presented in Section 4, and Section 5 concludes the research contribution and suggests future research directions. Although our use of the KITTI dataset primarily establishes the use of AEPF for automated driving applications, we contend that it can be used for other applications, such as robotics, drone navigation, and augmented reality.

## 2. Related Work

Three-dimensional object detection methods can be classified into three types: camera (or stereo)-based, LiDAR-based, and multi-modal fusion-based. As camera (or stereo) and LiDAR are the most common sensor setups for 3D object perception, the focus will be on methods involving these two technologies.

### 2.1. 3D Object Detection Using Images

Given the success of 2D detection methods in regressing 2D boxes in images, a straightforward approach to extend this paradigm to 3D detection is to just directly regress 3D localization parameters using a convolutional neural network (CNN). The shift from 2D to 3D detection involves utilizing the feature extraction capabilities of CNNs and extending them to accommodate the additional (albeit missing from individual camera images) spatial dimension present in 3D data. For instance, approaches such as that in [5,26,27] predict 3D bounding boxes using images as the sole input. These methods usually involve creating specialized loss functions to guide the learning of 3D parameters effectively and designing architectures that can capture essential depth cues and contextual information. Stereo-based methods [28,29] detect 3D objects from pairs of images, leveraging the additional geometric information from stereo images to infer depth using a disparity map. Since RGB images lack inherent depth information, methods like those in [30,31] perform depth estimation and generate pseudo-LiDAR representations for 3D object detection. With recent advancements in transformer-based architectures [32], researchers [33,34] have utilized 3D object queries and 3D–2D correspondence for 3D object detection. Furthermore, techniques like incremental structure-from-motion [35] and machine-learning-based image translation methods [36] have been developed to improve 3D point cloud reconstruction from RGB data providing spatial information that images alone cannot offer. Although these advancements in 3D structure recovery from images are crucial for 3D object detection and localization, LiDAR sensor data typically provides more accurate 3D information as a point cloud without requiring additional processing. Given the challenges of accurate depth estimation from images, LiDAR-based 3D object detectors tend to outperform all image-only methods for 3D object detection.

### 2.2. 3D Object Detection Using Point Clouds

Thanks to the direct depth information provided by LiDAR, point-cloud-based 3D object detectors have been the primary focus in recent years. A challenge with LiDAR data is that in its original representation, the point cloud contains sparse unordered points, which means that it cannot serve as an input to convolutional layers. However, a key advantage of LiDAR-based 3D object detection over multi-modal fusion-based methods is that these models do not require multi-sensor calibration and alignment. LiDAR-based 3D object detectors do not perform well for longer-distance objects. These methods can be distinguished by how they encode raw LiDAR data to extract features from the point cloud and can be categorized into point-based and grid-based methods.

In the point-based category, PointRCNN [37] utilizes the original point cloud data and employs PointNet++ [38] to learn per-point features to generate 3D proposals and segmentation masks. 3DSSD [39] increases network inference speed by replacing feature propagation and refinement modules with fusion sampling and candidate generation layers.

VoxelNet [8], a grid-based approach, utilizes voxelization to encode the raw point cloud data into fixed-size voxels to employ 3D CNNs to learn voxel features for classification and bounding box regression. SECOND [9] upgrades the original VoxelNet [8] approach by introducing sparse 3D CNNs to accommodate the sparse structure of point cloud data while significantly improving inference time. Pointpillars [7] adopts PointNets [40] as an encoder and organizes point clouds in vertical columns (pillars), which is processed by a 2D CNN detection head to perform 3D object detection, enabling even slower inference time than [9]. Overall, we observe a tradeoff between accuracy and runtime that influences the choice of method. These findings have prompted researchers to investigate alternative multi-modal fusion methods.

### 2.3. 3D Object Detection Using Multi-Modal Fusion Methods

While examining several 3D object detectors in popular detection benchmarks [23,24], most LiDAR-based methods surpass fusion-based methods because a significant amount of the objects measured are cars, whose sizes are often larger than cyclists and pedestrians. Several comparisons on smaller objects show that fusion-based methods do not perform worse than LiDAR-based methods [14]. Combining two modalities comes with an additional computational load of processing additional sensor information, which limits fusion-based methods to limit the number of convolutional operations. One of the earliest fusion methods, MV3D [12], transforms the point cloud into a BEV representation and a front view representation, then fuses this data with RBG image information. It begins by generating 3D proposals on the BEV feature map, projects these proposals onto the other two feature maps, and ultimately fuses region-based features to make the final prediction. AVOD [13] extracts features from RGB images and BEV before maps to fuse them for 3D object proposal generation. Conversion of point cloud data into 3D representation as BEV and front-view representation loses spatial information in a point cloud.

Cascaded-fusion methods [41,42] narrow regions for 3D data processing within a point cloud using information from 2D detectors. For example, Frustum Pointnet [43] uses a frustum-based methodology, where 2D proposals are lifted into 3D spaces using a frustum. The major drawback, however, is that these methods rely heavily on the 2D object proposal generation stage and would perform poorly in cases where 2D object proposal generation fails.

Late-fusion method, CLOCs [14] utilizes object candidates fusion to fuse 2D and 3D object detection candidates to exploit geometric and semantic consistency between 2D and 3D detections. Fast-CLOCs [15] uses a 3D detector-cued 2D image detector to reduce memory and computational load from the original CLOCs [14] implementation. Although these late-fusion methods perform well in benchmark detection tasks [23,24], the intermediate features and representations from images and point clouds are not correlated, which leads to loss of valuable contextual information captured by one sensor, which may not be effectively complemented by another sensor.

MVX-Net [18] proposes two fusion strategies—point-level and voxel-level—to fuse image and voxel features early. PointFusion [42] correlates LiDAR points with image features by projecting each point onto the image using the calibration matrix to obtain point-wise image features. Inspired by recent success in attention-based mechanisms [32] in focusing essential features, we extend the PointFusion approach by incorporating an attention mechanism to highlight important point-wise image features and voxel features before fusion to obtain 3D bounding boxes.

### 2.4. Research Gaps

Although methods like MVXNet [18] and PointFusion [42] fuse image and point cloud features early, these methods fail to highlight features from individual modalities. A recent work, AVFP-MVX [44], uses an attention mechanism within the 2D feature extraction module and processes the fused representation with Voxel-FPN. While this method uses attention to highlight essential features from images, it does not account for the most prominent point cloud features pre-fusion, as the attention mechanism is absent in the point feature extraction module. As existing voxel-based 3D object detectors that solely use LiDAR data [9,11,45] demonstrate strong performance, the current literature lacks methods that focus on the most prominent voxel features before fusion with image features such that the fused structure can be processed with any voxel-based 3D backbone. In summary, existing 3D object detection methods fail to emphasize prominent complementary features from image and voxel data before feature fusion. To the best of our knowledge, AEPF is the first attempt to emphasize voxel and image features separately before their fusion with two different techniques, ensuring that complementary features are effectively highlighted.

## 3. Proposed Fusion Methodology

Herein, two AEPF variants are presented to fuse images and point cloud information for 3D object detection, as shown in Figure 1. AEPF-S employs attention mechanisms for both image and point cloud features before fusion, while AEPF-L utilizes multi-head self-attention and uses image features to highlight important point cloud features before fusion. For both networks, the first stage involves feature extraction from 2D images. Post-feature extraction, following [18,42], points from the LiDAR point cloud are projected onto the camera image to obtain point-wise image features. Point-wise image and voxel features are highlighted with attention mechanisms for each before the feature fusion step. After feature fusion, a 3D backbone is used to regress bounding boxes from the multi-modal features. Each major step of AEPF is described in the following sections.

### 3.1. 2D Image Feature Extraction

The first step for AEPF involves feature extraction from 2D RGB images as shown in Figure 1. CNNs have been proven effective at extracting semantic information from images [3]. ResNet [46] (Residual Network) is a pioneering work in 2D computer vision that uses residual blocks with skip connections for improved feature extraction. Residual learning, introduced in [46], addresses information losses and gradient explosion issues in traditional CNNs. ResNet architectures are designed to balance network depth and computational efficiency. While ResNet-18 and Resnet-34 perform with limited computational resources, they are relatively shallow for complex feature extraction. With much greater depth layers on ResNet-101 and ResNet-152 variants, feature extraction with deeper layers adds extra computational cost, thereby making them unsuitable for real-time inference given current typical computational resources. Desiring a balance between depth and computational efficiency, ResNet-50 was used for feature extraction for both network variants proposed.

The 50-layered ResNet is categorized into four stages, each containing several residual blocks, which generate feature maps with channel sizes of [256,512,1024,2048], respectively. Since the first stage captures basic features like edges and textures, the batch normalization layers and parameters for this stage were frozen to help stabilize the training process. For the first network variant, AEPF-Small (AEPF-S), hierarchical feature maps from the second and third stages from backbone ResNet-50 were used for point cloud projection to obtain point-wise image features. For the second network variant, AEPF-Large (AEPF-L), we add a feature pyramid network (FPN) that takes feature maps from outputs of all ResNet stages to construct a pyramid of feature maps, allowing the capture of multi-scale information for object detection.

Post-feature extraction for both network variants, each 3D point from the LiDAR point cloud is projected onto the image using a known calibration matrix. We use a similar approach as [18,42] to attach the corresponding image feature to each 3D point. As feature connection happens early, both network variants can learn and summarize joint multi-modal representation for accurately regressing 3D bounding boxes.

### 3.2. Point Cloud Voxelization

After obtaining point-wise image features, we extract point-wise voxel features to enable fusion in the subsequent step. To manage the sparse and unstructured nature of the point cloud, the point cloud can be divided into equally spaced voxels to allow grid-based convolutional operations, as shown in Figure 1. VoxelNet [8] introduced a voxel feature encoding (VFE) layer to encode raw point clouds at individual voxel levels. A raw point cloud can be transformed into a 3D space divided into equally spaced voxels. A point in voxel is represented as: $P_{i}=\left\{x_{i},y_{i},z_{i},I_{i},c_{x},c_{y},c_{z}\right\}$, where $\left\{x_{i},y_{i},z_{i}\right\}$ are the XYZ coordinates of the point with intensity $I_{i}$ and $\left\{c_{x},c_{y},c_{z}\right\}$ represents the centroid of the voxel at which $P_{i}$ is located. For both network variants, we use dynamic voxelization [47], which establishes a bi-directional relationship between points and voxels laying the foundation for cross-view feature fusion.

The stacks of VFE layers containing fully connected networks (FCNs) transform the original point cloud into high-dimensional voxel features. Both network variants use the same voxel size, but the feature encoder for AEPF-S has 2 layers with 32 channels each and outputs point cloud features with 32 channels before fusion. The feature encoder for AEPF-L has two layers with 32 channels each and outputs point cloud features with 64 channels before fusion.

### 3.3. Attention Mechanisms and Point Fusion

After extracting point-wise image and voxel features, it is necessary to highlight the most important features from both image and voxel data before fusion. Attention-based mechanisms [32] are introduced to enhance fusion between the voxel and point-wise image features, as shown in Figure 1. Using an attention-based mechanism, different weights are assigned to different features, ensuring that the most essential features for fusion have a more significant impact on the final output. The selective weighting mechanism forces the model to focus on the most important features to improve object detection performance and plays a crucial role in addressing common issues such as false positives and false negatives. By assigning higher attention weights to prominent object features, the model can detect objects that might otherwise be missed, thus reducing false negatives. Conversely, the attention mechanism diminishes the impact of less relevant features, which helps minimize false positives and prevent incorrect classifications. In cluttered or complex environments, this focused approach ensures that only the most significant features are considered for detection, thereby improving the accuracy and reliability of the detection model.

For AEPF-S, a linear layer transforms the input point-wise image features followed by a rectified linear unit (ReLU) activation as shown in Figure 1. An additional linear layer produces a single scale value as the attention weight for each point-wise image feature. The same transformation process is applied to the voxel features to produce attention weights.

The attention scores calculated are used to weigh the original features as:

$$F_{\mathrm{img}}^{\mathrm{att}}=F_{\mathrm{img}}⊙\;\mathrm{ReLU}\left(A_{\mathrm{img}}\right)$$

$$F_{\mathrm{vxl}}^{\mathrm{att}}=F_{\mathrm{vxl}}⊙\;\mathrm{ReLU}\left(A_{\mathrm{vxl}}\right)$$

where $F_{\mathrm{img}}\in R^{N\times D_{\mathrm{img}}}$ denotes point-wise image features, $F_{\mathrm{vxl}}\in R^{N\times D_{\mathrm{vxl}}}$ denotes voxel features, $F_{\mathrm{img}}^{\mathrm{att}}$ and $F_{\mathrm{vxl}}^{\mathrm{att}}$ denote attended image and voxel features, respectively, $N,D_{img}$, and $D_{vxl}$ represent the number of features, the dimension of input image features, and the dimension of input voxel features, respectively, and $A_{\mathrm{img}}$ and $A_{\mathrm{vxl}}$ contains attention scores for image features and voxel features representing their importance. Weighted features after attention are concatenated for fused feature representation for AEPF-S, $F_{\mathrm{fused}}^{S}$:

$$F_{\mathrm{fused}}^{S}=F_{\mathrm{img}}^{\mathrm{att}}+F_{\mathrm{vxl}}^{\mathrm{att}}$$

AEPF-L employs a multi-headed self-attention (MHSA) [32], as shown in Figure 1, to enhance the most significant voxel features using information from the image features before fusion. MHSA extends the self-attention process across multiple parallel heads, each with learnable parameters to produce query, key, and value vectors. Queries represent the features being focused on, keys represent the features against which the queries are compared, and values contain the information that is aggregated based on the attention weights. For AEPF-L, point-wise image features are used as queries as they capture rich semantic information which helps in identifying relevant point-wise voxel features. Point-wise voxel features are used as keys and help in determining the relevance of each voxel feature in relation to the query. Values are also derived from voxel features and are used to update the voxel features based on the weights determined by the attention mechanism. Comparing queries with keys, the MHSA computes attention weights indicating the significance of each voxel feature and applies attention to the values (point-wise voxel features). Point-wise image and voxel features are linearly transformed into queries (Q), keys (K), and values (V) as:

$$Q={\mathrm{Linear}}_{Q}\left(F_{\mathrm{img}}\right)\;\mathrm{reshaped}\;\mathrm{to}\;{\left(B,H,-1\right)}^{T}$$

$$K={\mathrm{Linear}}_{K}\left(F_{\mathrm{vxl}}\right)\;\mathrm{reshaped}\;\mathrm{to}\;{\left(B,H,-1\right)}^{T}$$

$$V={\mathrm{Linear}}_{V}\left(F_{\mathrm{vxl}}\right)\;\mathrm{reshaped}\;\mathrm{to}\;{\left(B,H,-1\right)}^{T}$$

where *B* and *H* denote the batch size and number of attention heads, respectively. The attention weights (A) are computed using the scaled dot product attention mechanism [32] as:

$$A=\mathrm{softmax}\left(\frac{Q\cdot K^{T}}{\sqrt{D_{k}}}\right)$$

where $D_{k}$ is the dimension of the keys. The attention weights are applied to the values to obtain attended values, Z:

Z=A·V

The combined attention heads are transformed to produce the final attended voxel features:

$$F_{\mathrm{vxl}}^{\mathrm{att}}={\mathrm{Linear}}_{O}\left(Z\right)$$

Final attended voxel features are concatenated with point-wise image features for fused feature representation for AEPF-L, $F_{\mathrm{fused}}^{L}$:

$$F_{\mathrm{fused}}^{L}=F_{\mathrm{img}}+F_{\mathrm{vxl}}^{\mathrm{att}}$$

### 3.4. 3D Backbone Network

Post-point-wise image and voxel feature fusion, fused feature representation must be passed through a 3D backbone network for 3D object detection as shown in Figure 1. Fused feature representation for both network variants can be passed through any voxel-based 3D backbone networks [7,9,37,45]. SECOND [9] introduced 3D sparse convolution to handle sparse LiDAR point clouds. We use the single-stage SECOND [9] backbone for both of our network variants because of its computational efficiency and effectiveness in handling sparse data over other counterparts. The voxel structure to be fed into the SECOND-based 3D backbone network consists of 1600×1408×40 voxel grids with each voxel of size 0.05×0.05×0.1 m for both network variants.

AEPF-S processes 128 input channels in the SECOND backbone with two stages, each consisting of five layers. AEPF-L processes 258 input channels from the SECOND backbone to handle richer feature representations from the fused representation. Both network variants also output higher dimensional feature maps (128 and 256 for AEPF-S and AEPF-L, respectively) using the FPN from SECOND to enhance multi-scale representation.

Since we use the SECOND backbone, we follow the same multitask loss function used in [9] which is a combination of classification loss ($L_{cls}$), regression loss ($L_{reg}$), and direction classification loss ($L_{dir}$):

$$L_{\mathrm{total}}=\beta_{1}L_{cls}+\beta_{2}L_{reg}+\beta_{3}L_{dir}$$

where $\beta_{1}$, $\beta_{2}$, and $\beta_{3}$ are the weights for classification loss, regression loss, and direction classification loss, respectively. We set $\beta_{1}=2.5$, $\beta_{2}=1$, and $\beta_{3}=0.2$ for training both AEPF variants. The choice of these weights was determined through extensive experimentation by systematically varying the weights for each component to assess their impact on model performance. We also follow [9] in parametrizing 3D ground truth boxes and 3D anchors.

## 4. Experimental Validation

### 4.1. Dataset

The proposed AEPF method is evaluated on KITTI Vision Benchmark [23], which provides 7481 training samples and 7518 testing samples for the 3D and birds-eye view (BEV) object detection tasks. The difference between 3D and BEV object detection tasks is that BEV does not consider the object’s height. Ground truth labels are provided for the 7481 training samples, and testing samples are evaluated by submitting results to the online KITTI server [23]. Each sample contains a LiDAR point cloud, corresponding RGB image, and their calibration parameters. The dataset categorizes object detection tasks into three difficulty levels—“easy”, “moderate”, and “hard”—based on fully visible and slight truncation, partly occluded and moderate truncation, and challenging to see and severe truncation, respectively.

### 4.2. Training Configuration

The range of point cloud data was limited to [0,70.4]×[−40,40]×[−3,1] meters in (x,y,z) axes to remove points outside of detection range. Following [48], the training data was divided into train and validation splits containing 3712 and 3769 frames, respectively. The three classes aimed for detection are cars, pedestrians, and cyclists. We use the same data augmentation techniques described in [49].

### 4.3. Training Settings

Both networks were trained on a single NVIDIA A6000 GPU with the ADAM optimizer. The total batch size was set to 6, and the cosine annealing strategy was used to adjust the learning rates dynamically. This scheduler decreases the learning rate following a cosine curve, starting at 0.0003 and reducing it to a minimum of 0.0001.

### 4.4. Evaluation Metrics

KITTI uses average precision (AP) for 3D object detection and BEV detection to evaluate each category within each difficulty level, calculated with 40 recall positions. For multi-class evaluation across multiple difficulty levels, we use mAP as the evaluation metric, the mean AP of all categories across all difficulty levels. The IoU thresholds for this metric for cars, pedestrians, and cyclists are 0.7, 0.5, and 0.5, respectively, as suggested in the KITTI [23] server. Predictions are considered correct when the IoU of the predicted bounding box and ground-truth box exceeds those thresholds.

### 4.5. Results

Table 1 shows the validation results for our methods. It compares them against LiDAR-based detection methods as well as LiDAR and image-based 3D detection methods. We did not include image-only methods in our comparison because LiDAR and fusion-based methods consistently outperform image-only methods in 3D object detection tasks. For LiDAR-based methods, we specifically chose voxel-based methods, since the fused representation for both AEPF variants can be processed with any voxel-based 3D backbone. The proposed fusion techniques achieved improved performance compared to the original SECOND [9] method with improved AP scores across all categories ranging from +0.06 to +7.05. AEPF-L outperforms the MVXNet [18] method, which also employs the PointFusion [42] strategy, achieving improved AP scores across all categories, with increases ranging from +3.49 to +8.75. The BEV mAP score for car detection in the easy category was the highest among other camera and LiDAR fusion-based methods, with AEPF-L scoring 95.27 and AEPF-S coming in second at 94.40. When comparing fusion-based methods, AEPF-L demonstrated the second-highest AP score for 3D car detection in all categories, just below CLOCs [14], which uses a late-fusion strategy that combines detection candidates from PV-RCNN (LiDAR) and Cascade R-CNN [50] (image), making it more computationally expensive than AEPF-L. Although the score for the easy category for 3D car detection was close to CLOCs (−1.65), AP scores for the moderate and hard categories for both detection variants were significantly lower than CLOCs, with differences ranging from −6.88 to −9.80. This could be addressed in future work by thoroughly exploring advanced attention-enabled fusion strategies to improve performance across all detection categories. Fused point-wise image features and voxel features for AEPF can be processed with any voxel-based 3D backbone which allows AEPF-based networks to swap the existing single-stage SECOND-based 3D backbone to other multi-stage 3D backbones like Part-A2 [51], Voxel-RCNN [45], and PointRCNN [37] for tasks that require greater accuracy in the expense of computational resources. Given the strong evidence of accuracy improvements over the baseline SECOND when using a SECOND-based 3D backbone, we argue that employing a double-stage 3D backbone network, similar to the LiDAR-only methods in Table 1 [11,45,52], would result in better accuracy for AEPF-based methods compared to those methods.

**Figure 2:**
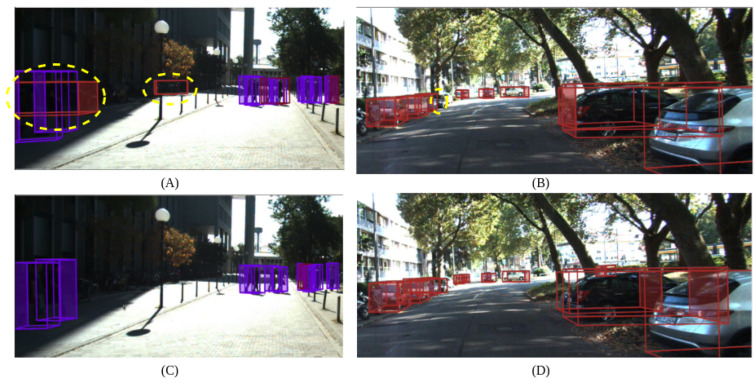
Visualization of detection results for two AEPF variants. Panels (**A**,**B**) display results for AEPF-S, while panels (**C**,**D**) show results for AEPF-L. False positives and missed detections from AEPF-S, highlighted by dotted yellow lines, are effectively addressed by AEPF-L. Red bounding boxes indicate cars and purple bounding boxes indicate pedestrians.

Qualitative results for both detection variants are displayed in Figure 2. The 3D object detection outcomes, based on image and point cloud data, are projected onto the image for visualization. AEPF-L successfully addressed the false positives and missed detections in Figure 2A,B from AEPF-S shown in Figure 2C,D, reinforcing the rationale behind proposing two variants: one optimized for inference speed and the other for improved accuracy.

**Figure 3:**
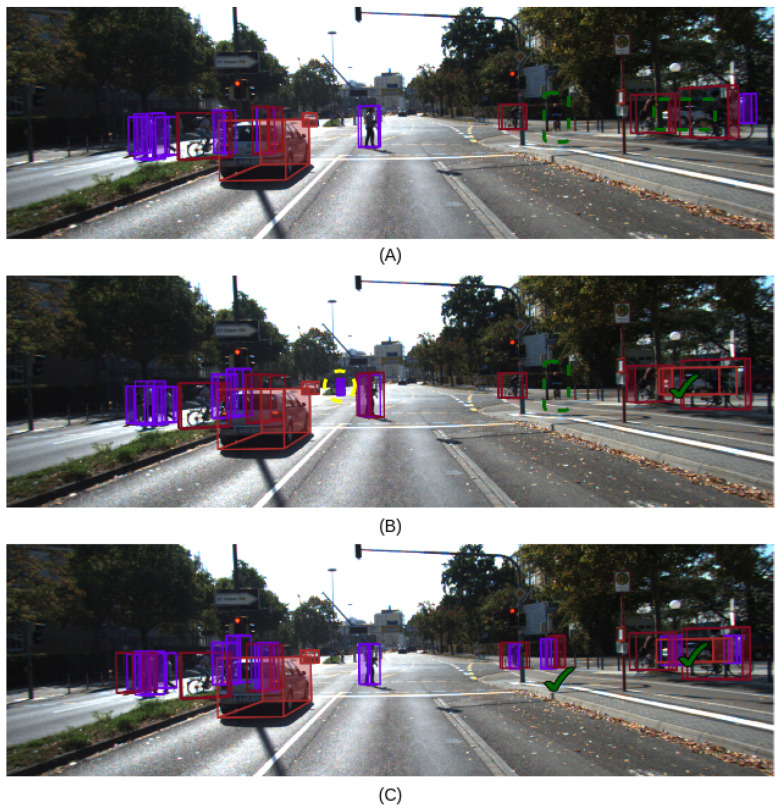
Visualization of detection results for (**A**) MVXNet (obtained from [53]), (**B**) AEPF-S, and (**C**) AEPF-L. Dotted green lines indicate false negatives, while dotted yellow lines indicate false positives. AEPF-L effectively resolves false negatives identified by MVXNet and AEPF-S. Purple bounding boxes indicate pedestrians and red bounding boxes indicate cars.

Additionally, AEPF-S, AEPF-L, and an early feature fusion method (MVXNet [18]) with similar backbone configuration were run on the same machine for a fair comparison, and the results are shown in Table 2. We chose to compare our approach with MVXNet [18], as it was readily available for implementation [53] and shares architectural similarities with our method. Notably, AEPF-S demonstrated enhanced inference times compared to the baseline MVXNet and AEPF-L. AEPF-S achieved a significant improvement in inference speed, exceeding MVXNet by +4.8 fps. This suggests that the attention mechanism used for AEFP-S enabled us to use a more straightforward configuration while maintaining the accuracy of other fusion-based methods. Additionally, AEPF-L outperformed MVXNet in terms of mAP scores, outperforming it by +1.5 in 3D detection and +0.32 in BEV detection despite the slightly slower inference speed (−1.2 fps), all while keeping the same 2D and 3D backbone configurations. This suggests that AEPF-L’s attention mechanisms significantly improve detection performance at the expense of only a minor increase in inference time. This becomes particularly evident in scenes with numerous pedestrians, cyclists, and cars. Figure 3 illustrates detection results in a scene containing multiple pedestrians, cyclists, and cars. Due to the limited training data for pedestrians and cyclists, both MVXNet and AEPF-S fail to detect a cyclist, as shown in Figure 3A,B. In contrast, AEPF-L, with its attention mechanism successfully detects the cyclist as shown in Figure 3C. AEPF-based detection frameworks also work well in cluttered environments; for instance, where MVXNet fails to detect a car amidst object clutter, the AEPF-based methods accurately identify it, as shown in Figure 3. These results further demonstrate the effectiveness of attention mechanisms within AEPF-based networks for accurate object detection.

### 4.6. Ablation Studies

To evaluate the contribution of specific components in the proposed detection pipeline, we conducted ablation experiments for AEPF-S and AEPF-L. Given the need for AEPF-S to infer at faster speeds, the computationally expensive part lies in the image feature extraction process, specifically from ResNet-50. To determine which stages of features are most critical, we compared three AEPF-S variants, each extracting features from different ResNet stages, as shown in Table 3. For baseline comparison, we also included results from a fusion procedure that uses features from all stages without applying any attention mechanism. The results showed that using features from stages 2 and 3 with the AEPF-S attention mechanism showed the best performance, with an improvement of +3.81 in Car 3D mAP and +1.96 in Car BEV mAP compared to the baseline.

We also performed ablation experiments to evaluate the impact of the number of attention heads in the attention mechanism used for AEPF-L. We tested three different settings with the number of attention heads set to 4, 8, and 12. As shown in Table 4, AEPF-L achieved the best results when the number of attention heads was set to 4. The best performance of AEPF-L with four attention heads can be attributed to a balance between model complexity and capacity, allowing it to capture essential features without overfitting. Moreover, using fewer attention heads improves computational resource utilization, reducing redundant feature extraction and highlighting important point cloud features for more focused learning.

## 5. Conclusions

This paper introduced a novel multi-modal and multi-class 3D object detection framework named Attention-Enabled Point Fusion (AEPF), which leverages an attention mechanism to fuse features from images and point clouds, thereby enhancing the accuracy of 3D object detection compared to traditional methods. Our results highlight the potential of early feature fusion and attention mechanisms in enhancing 3D object detection. Through extensive experiments on the KITTI dataset, the effectiveness of our method was validated, showcasing competitive results in both 3D and BEV object detection tasks across different difficulty levels.

Two model variants, AEPF-S and AEPF-L, are proposed, each tailored to different speed and accuracy trade-offs, providing flexibility for various application needs. AEPF-S is designed for scenarios that demand faster inference speeds. It is ideal for immediate real-time applications with other functional oversight (e.g., a human driver in advanced driving assistance systems) and is hardware-limited. Conversely, AEPF-L prioritizes higher accuracy, making it well-suited for limited oversight critical tasks such as autonomous driving, where safety is paramount. The complexity of the AEPF framework is effectively managed by introducing two variants tailored to different computational needs, thereby ensuring efficient resource utilization while delivering SOTA performance. The trade-offs between inference speed and detection accuracy were thoroughly analyzed. AEPF-S achieved significantly higher inference speeds, making it particularly appealing for resource-constrained environments while maintaining SOTA accuracy. AEPF-L, although inferencing 4.6% slower than the compared baseline, provided substantial improvements in detection performance (+1.63 mAP in car 3D detection and +0.49 mAP in car BEV detection), making it ideal for applications where accuracy is critical, even at the expense of increased computational demands.

Future work will be aimed at investigating the scalability and adaptability of these models, including a more exhaustive analysis across varying lighting conditions, noise levels, and other challenging scenarios to further evaluate and enhance the robustness of the AEPF variants. This will involve further refinement of attention mechanisms to enhance detection accuracy while satisfying low computational demands, and exploring hybrid strategies that dynamically switch between AEPF-S and AEPF-L based on real-time assessments of the environment and available compute resources. Furthermore, the modularity of the AEPF framework also allows for the integration of advanced multi-stage networks as the fused point-wise image and voxel features can be processed with any voxel-based 3D backbone networks based on task severity with minimal customization. This preliminary work into AEPF is promising, and further exploration could make the model scalable to larger datasets and adaptable for real-time applications in robotics, navigation, and autonomous driving.

## Figures and Tables

**Table 1:** Car 3D detection results on the KITTI validation set. We use [9] for baseline comparison. Cells are left blank for methods that did not report their validation statistics in their paper. The best and second best performance among fusion-based methods only for every category is highlighted in black and blue, respectively.

Method	Modality	Car 3D AP (R40)			Car BEV AP (R40)		
		Easy	Mod.	Hard	Easy	Mod.	Hard
VoxelNet [8]	LiDAR	81.97	65.46	62.85	89.60	84.81	78.57
SECOND [9] (Baseline)	LiDAR	87.43	76.48	69.10	89.96	87.07	79.66
PointPillars [7]	LiDAR	86.46	77.28	74.65	-	-	-
PointRCNN [37]	LiDAR	88.72	78.61	77.82	-	-	-
PV-RCNN [11]	LiDAR	92.57	84.83	82.69	95.76	91.11	88.93
Voxel-RCNN [45]	LiDAR	92.38	85.29	82.86	95.52	91.25	88.99
CT3D [52]	LiDAR	92.85	85.82	83.46	96.14	91.88	89.63
MV3D [12]	LiDAR+RGB	71.29	62.68	56.56	86.55	78.10	76.67
F-PointNet [43]	LiDAR+RGB	83.76	70.92	63.65	88.16	84.02	76.44
CLOCs [14]	LiDAR+RGB	**92.78**	**85.94**	**83.25**	93.48	**91.98**	**89.48**
MVXNet [18]	LiDAR+RGB	85.50	73.30	67.40	89.50	84.9	79.00
AEPF-S (Ours)	LiDAR+RGB	89.87	77.83	73.45	**94.40**	87.13	84.74
AEPF-L (Ours)	LiDAR+RGB	**91.13**	**79.06**	**76.15**	**95.27**	**88.39**	**85.91**

**Table 2:** Comparison of three different methods for 3D object detection. We used open-sourced implementation in MMDetection3D [53] for ResNet-50 and SECOND-FPN-configured MVXNet [18]. The metrics for the top-performing model in each category are highlighted in bold.

Method	Modality	Backbone Configuration		Speed (fps)	Car 3D mAP	Car BEV mAP
		2D	3D			
MVXNet [18] (Baseline)	LiDAR+RGB	ResNet-50 + FPN	SECOND + FPN	26.2	80.48	89.37
AEPF-S (Ours)	LiDAR+RGB	ResNet-50	SECOND + FPN	**31.0**	80.38	88.75
AEPF-L (Ours)	LiDAR+RGB	ResNet-50 + FPN	SECOND + FPN	25.0	**82.11**	**89.86**

**Table 3:** Ablation experiments to choose feature extraction pipeline for AEPF-S before fusion. Features from stages 2 and 3 were used without an FPN for the final AEPF-S architecture. The metrics for the top-performing configuration in each category are highlighted in bold.

ResNet-50 Stages	Attention	Car 3D mAP	Car BEV mAP
All	-	76.57	86.79
1 and 2	AEPF-S	79.37	87.31
2 and 3	AEPF-S	**80.38**	**88.75**
3 and 4	AEPF-S	79.58	87.54

**Table 4:** Ablation experiments to choose the number of attention heads for MHSA in AEPF-L. For the final model, the number of heads was set to 4. The metrics for the top-performing configuration in each category are highlighted in bold.

Num. of Heads	Attention	Car 3D mAP	Car BEV mAP
4	AEPF-L	**82.11**	**89.86**
8	AEPF-L	81.54	89.47
12	AEPF-L	80.06	88.82

## Data Availability

Data used for this research are publicly available at https://www.cvlibs.net/datasets/kitti/index.php (accessed on 15 April 2024).
